# Developmental Trajectories of Conduct Problems and Cumulative Risk from Early Childhood to Adolescence

**DOI:** 10.1007/s10964-018-0971-x

**Published:** 2019-02-01

**Authors:** Leslie Morrison Gutman, Heather Joshi, Ingrid Schoon

**Affiliations:** 1 University College London, 1-19 Torrington Place, London, WC1E 7HB UK; 2 UCL Institute of Education, 20 Bedford Way, London, WC1H 0AL UK

**Keywords:** Conduct problems, Developmental trajectories, Cumulative risk, Co-morbidity, Group-based modeling

## Abstract

Pathways into and out of conduct problems differ by circumstances experienced since infancy. There is a research gap in understanding how these developmental patterns vary according to the timing and persistence of risk and whether there are differences across ecological domains. This study examines variations in trajectories of conduct problems between ages 3 to 14 years and associated child, family and socio-economic risk factors from ages 9 months to 14 years, drawing on the UK Millennium Cohort Study (*n* = 17,206, 49% female), a nationally representative longitudinal study of children born between 2000/02. Group-based modeling was used to identify four distinct trajectories of conduct problems: low (56%), persistent high (8%), childhood-limited (23%) and adolescent-onset (13%). All three problem pathways were associated with high levels of exposure to risk, particularly early socio-economic and persisting child and family risks. However, while for the persistent and adolescent-onset pathways, exposure to higher levels of family and child risks continued through adolescence, it receded for the childhood-limited trajectory. The effects of early socio-economic disadvantage persisted for those on the adolescent-onset trajectory, highlighting the importance of early markers for this later onset group. Maternal smoking in pregnancy continued to be a significant predictor for all three conduct problem groups, even up to age 14 years. The findings indicate that different influences and processes may explain diverse pathways of conduct problems. This offers insights into who and what might be targeted and when might be the most effective developmental window for intervention.

## Introduction

Conduct problems refer to a repetitive pattern of behavior that violates the rights of others or age-appropriate norms or rules (American Psychiatric Association 2013). Children experience different pathways of conduct problems based on the age of onset, developmental course and associated risk factors (Frick and Viding 2009; Moffitt 2006). Despite the importance of early intervention, few studies have examined conduct problem trajectories and their associated risk factors from the youngest age that conduct problems can be measured reliably, which is age 3 (Shaw 2013). There are particular gaps in the research on how developmental patterns of conduct problems vary according to the timing and persistence of risk and whether these differ across ecological domains, such as child, family and socio-economic factors. For example, does the timing of exposure to risk differ across diverse trajectories of conduct problems? Does later exposure to risk matter over and above early exposure? Are there differential effects of risks at the level of family (e.g., parental depressive symptoms) compared to socio-economic (e.g., low-income) or child-level (i.e. raised hyperactivity or low verbal skills) risks?

A better understanding of how different developmental disruptions influence the pathways of conduct problems is critical for designing interventions that enhance a child’s development and reduce their disruptive behavior, even if the risk factors themselves are unchangeable (Frick 2012). Such information is essential in tailoring interventions based on distinct conduct problem pathways and individual mechanisms of change. This study aims to fill these gaps by identifying trajectories of conduct problems from ages 3 to 14 years and examining the role of timing and persistence of risk across different domains from ages 9 months to 14 years, using a nationally representative sample of children living in the UK.

### Developmental Trajectories of Conduct Problems

A developmental-pathways approach provides a framework to comprehend the progression of conduct problems as well as the mechanisms underlying its onset and continuity (Frick 2012). In one such approach, Moffitt (1993) identified two groups of children based on the age of onset and developmental course of conduct problems, differentiating between “life-course, persistent” and “adolescent-limited” trajectories. Those following a life-course, persistent pathway show consistent and elevated levels of conduct problems from early childhood (i.e., before 10-years-old), exhibiting antisocial behavior through adolescence and adulthood (Broidy et al. 2003; Frick and Viding 2009). According to Moffitt (1993, 2006), this pathway may be explained, in part, by neuropsychological deficits such as hyperactivity, impulsiveness and inattention; difficult temperament and poor verbal abilities, which reciprocally interact with disruptive family and social relationships, poor parenting and other stressors.

Adolescent conduct problems, on the other hand, supposedly manifest between ages 10 to 17 years. Most on the adolescent pathway have been thought to mature out of their antisocial behavior in preparation for adulthood; thus their trajectory has been described as “adolescent-limited” (Moffitt 2006; Odgers et al. 2008). However, adolescent conduct problems are not necessarily limited to adolescence and may be a marker for co-morbid mental health problems, antisocial behavior and later criminality (Fairchild et al. 2013; Kretschmer et al. 2014; Sentse et al. 2017). Thus, the term “adolescent-limited” may be misleading and should instead be referred to as “adolescent-onset” (Sentse et al. 2017). Early risk factors for the adolescent-onset pathway include parental instability, low IQ and difficult temperament (Bor et al. 2004; Odgers et al. 2007). Those on this pathway often show greater rebelliousness to parents and other authority figures (Brezina and Piquero 2007), which combined with greater access to adult privileges, poor parental monitoring and association with problematic peers, lead to adolescent-onset conduct problems (Moffitt 2006; Odgers et al. 2008).

Among those with childhood-onset, many do not exhibit conduct problems later in development (Moffitt 2006). Group-based trajectory modeling has identified another trajectory termed “childhood-limited” or “early-onset, desisting”, characterised by high levels of conduct problems in early childhood, which recede in later childhood and adolescence. Similar to children in the persistent group, those on the childhood-limited pathway have been shown to have cognitive deficits (e.g., low verbal ability), disruptive developmental traits and reside in households with greater family instability, socio-economic deprivation and less effective parenting (Barker and Maughan 2009; Odgers et al. 2008). Although children in the persistent and childhood-limited pathways appear to share many early risks, their trajectories are differentiated by the range and severity of exposure to risk (Barker and Maughan 2009; Gutman et al. 2018; Odgers et al. 2008). Furthermore, while the childhood-limited group have low levels of conduct problems in later childhood and adolescence, there is speculation that complete recovery rarely occurs (Bevilacqua et al. 2017). For example, previous studies find that this group shows higher levels of depressive symptoms in adolescence (Sentse et al. 2017), as well as internalizing problems, financial difficulties and smoking in adulthood (Odgers et al. 2008).

Numerous studies have supported the identification of these three conduct problem trajectories: persistent, adolescent-onset and childhood-limited, in addition to a low problem group (see Bevilacqua et al. 2017, for a review). These studies have varied across populations, age ranges and measures used to assess conduct problems (e.g., different versions of the Strengths and Difficulties Questionnaire (SDQ), Childhood Behavior Checklist (CBCL) or other measures). For example, drawing on the Dunedin Study (New Zealand), Odgers et al. (2008) used the DSM-IV-CD categories to assess conduct problems of children from ages 7 to 15 years. They identified four trajectory groups characterised by low-level conduct problems (56% males; 55% females), childhood-limited (23% males; 20% females), adolescent-onset (12% males; 17% females) and persistent problems (8% males; 7% females). Barker and Maughan (2009) examined data collected for the UK Avon Longitudinal Study using the SDQ to assess conduct problems at ages 4, 7, 8, 10, 12 and 13 years. They also identified four subgroups among males and females, comprising 64% of children with low-level conduct problems, 15% childhood-limited, 12% adolescent-onset and 9% early-onset, persisting problems. A more recent study on a Belgian population-based sample of 4 to 17 year-olds (Sentse et al. 2017) using maternal reports of the CBCL also identified four subgroups of conduct problem trajectories, labelled as low (48%; 40% boys), childhood-limited (12%; 52% boys), adolescence-onset (15%; 53% boys) and persistent (25%; 66% boys).

Despite an improved understanding of variations in conduct problem trajectories and their distinguishing risk factors, most of this research has focused on specific risk factors at one point in development (generally early childhood) or associated outcomes. What is lacking is a better understanding of the timing and persistence of exposure to risk and the accumulation of different risks over time. There is a dearth of evidence on whether specific risks persist or emerge across different ecological domains of development and how these potential changes shape conduct problem trajectories.

### Timing and Duration of Cumulative Risk

#### Cumulative risk

Developmental research has shown that risk factors do not occur in isolation but tend to co-occur. Economic hardship, for example, has been associated with increased parental distress and discord, as well as reduced capacity for parenting and greater risk for relationship break-up (Conger et al. 2010). The constellation of risks across domains is considered to be more important than any single factor alone (Evans et al. 2013; Schoon et al. 2003). Most previous research focusing on cumulative risk factors used a summative risk index, offering a single composite score (Emerson et al. 2011; Flouri 2008). The most common approach is to add up a set of dichotomized risk factors (1 = presence of risk; 0 = absence of risk) into a summative risk index. Dichotomization of important explanatory factors does not cause a decrease in their measured association with psychiatric and delinquency outcomes (Farrington and Loeber 2000) and allows the computation of a summative index of which there are multiple advantages, including reducing measurement error, enhancing validity by including multiple indicators and avoiding multi-collinearity (Evans et al. 2013). The assessment of cumulative exposure to risk provides a straightforward, easily interpretable way to identify children at increased odds for developing a range of maladaptive outcomes, which has important implications for targeted interventions.

Previous studies have shown that risk operates in a cumulative manner in predicting children’s behavior problems—the more risk children experience, the worse their problems (Appleyard et al. 2005; Atzaba-Poria et al. 2004). According to Bronfenbrenner (Bronfenbrenner and Evans 2000), human development involves interactions between the child and ecological levels of development seen as proximal to distal (i.e., individual, family, and the wider context). Cumulative exposure to risk is likely to disrupt these processes, which are necessary to support healthy development. Children may be able to handle high levels of cumulative risk in one context, if levels in other domains are relatively low, and if high levels are not persistent (Evans et al. 2013). Using a summative overall score gives equal weight to all risk factors, however, without taking into consideration the relative contribution of different domains of cumulative risk. There is growing awareness that processes which link risks to individual development operate at varying levels of specificity, and there is a need to distinguish between socio-economic disadvantage per se and other associated aspects of adversity, such as family structure or individual level capabilities (Ackerman et al. 1999; Fergusson and Lynskey 1996; Schoon et al. 2011). Previous research has shown that cumulative risk indices from different ecological domains (i.e., child, socio-cultural, family, and peer) predict children’s behavior problems (Deater-Deckard, Dodge, Bates, & Pettit, 1998). Further studies have elucidated the importance of proximal (e.g., involved parenting) factors versus more distal (e.g., low parental education or income) cumulative risk in predicting children’s externalizing behavior (Atzaba-Poria et al. 2004; Trentacosta et al. 2008). Similar research has yet to be undertaken, examining how ecological domains of cumulative risk predict developmental trajectories of conduct problems from early childhood to adolescence. This study thus uses multiple cumulative risk indices to assess the relative and independent contributions of cumulative risk in socio-economic, family and child-level domains on conduct problems trajectories.

#### Timing and Persistence of Risk

In addition, it is of critical importance to illuminate sensitive developmental windows for cumulative exposure to risk and to understand the effects of transient versus persistent exposure to risk on the development of conduct problems. Studies have examined the effects of cumulative risk on behavior outcomes, showing that early cumulative risk predicts later problematic behavior, even when cumulative risk in middle childhood has been taken into account (Appleyard et al. 2005). However, the association of cumulative risk with trajectories of conduct problems, particularly when considering their timing and persistence from early childhood to adolescence, has yet to be explored.

There is great diversity in the temporal dimension of exposure to risk: some risks might only be short term, although many last for most of the childhood years (Flouri and Kallis 2007). Moreover, early adversity might be overcome by improved circumstances, but may nevertheless leave the individual potentially more vulnerable to any disadvantage experienced at a later stage (Cicchetti and Aber 1998; Schoon et al. 2003). In addition, persistent adversity has generally stronger effects than intermittent or short term exposure (Ackerman et al. 1999). On the other hand, there is evidence to suggest that adversity experienced during early childhood, as opposed to later exposure, has a crucial impact on future adjustment (Duncan et al. 1998; Schoon et al. 2011), or that concurrent risk plays a crucial role (Campbell et al. 2000). The relative contributions of early versus later, or persistent effects, can only be elucidated by drawing on longitudinal data, which can provide detailed information about individuals followed over time.

## Current Study

Using the UK Millennium Cohort Study (MCS), the current study aims: first, to distinguish heterogeneous trajectories of conduct problems from early childhood to adolescence; and second, to examine how the timing and persistence of cumulative risk differentiate these trajectories. Using group-based trajectories, this study aims to identify distinct developmental trajectories of parent-reported conduct problems from ages 3 to 14 years. Clinically meaningful age cut-offs based on national norms are used, which have been shown to predict later conduct disorder (Meltzer et al. 2000). In line with other studies that have examined trajectories of conduct problems from early childhood (Barker and Maughan 2009; Odgers et al. 2008; Gutman et al. 2018; Sentse et al. 2017), four or more distinct conduct problems trajectories are expected, including persistent, childhood-limited, adolescent-onset and low pathways.

This study then investigates how these trajectories differ according to time-invariant factors (e.g., gender, ethnicity, teenage mother, smoking in pregnancy and low birth weight), and time-varying risk factors measured from ages 9 months to 14 years. To examine timing and persistence of cumulative risk, the same time-varying risk factors were required at each measurement, as much as possible. All the factors listed below were measured at each wave of the survey from ages 9 months to 14 years (with the exception of child risk factors and parental depressive symptoms which start at 3 years). Child factors include low verbal ability and co-morbid problems. Family factors comprise single or step-parent families, poor parental physical health, parental depressive symptoms and large family size. Socio-economic factors include low parental education, low income, parental worklessness and social housing.

Using the same cumulative risk scores for child, family and socio-economic domains at each time point, different assumptions are considered regarding the effects of timing (in terms of age of the child) and persistence (controlling for exposure to previous risk) on developmental trajectories of conduct problems. This study first examines the assumption of cumulative risk (Appleyard et al. 2005; Atzaba-Poria et al. 2004), expecting that persistent exposure to multiple risks across several time points generates long-term conduct problems (Masten et al. 2005; Masten and Cicchetti 2010). Second, according to the assumption of emerging psychopathology, evolving and current exposure to risk may provide a trigger for further adjustment problems (Cicchetti and Toth 2009; Rutter and Sroufe 2000). Trigger events can include adverse experiences, such as family break-up, parental unemployment or other unexpected life events. Third, in line with previous evidence, early risk factors, such as socio-economic disadvantage or family instability may show concurrent as well as later effects (also referred to as latency) on developmental trajectories, (Keiley et al. 2000; Schoon et al. 2003). Fourth, according to the assumption of maturation effects, many children grow out of initial problem behavior as they mature, differentiating between persistent and childhood-limited pathways (Barker and Maughan 2009; Moffitt 1993; Odgers et al. 2008; Sentse et al. 2017). A desisting pattern may be found, particularly among children with relatively low levels of exposure to risk or risk that does not persist over time.

## Method

### Study Sample

MCS is a nationwide multi-purpose longitudinal study following children born in all four countries of the UK between September 2000 and January 2002 (Joshi and Fitzsimons 2016). The survey has a complex clustered and disproportionately stratified design. The clusters were electoral wards, and the strata oversampled the three smaller countries of the UK (to allow for analysis within them), wards with high child poverty in all four countries and wards with high minority ethnic populations in England. Within a ward, all children born in the target period were eligible, regardless of birth order. Data are so far available from six waves of interviews with the families. The first survey, MCS1 (child age 9 months) was in the field mainly in 2001, MCS2 (age 3 years) in 2004, MCS3 (age 5 years) in 2006, MCS4 (age 7 years) in 2008, MCS5 (age 11 years) in 2012 when the cohort children were in their last year of primary school and MCS6 (age 14 years) in 2015 when they were in secondary school. Although an increasing part of each survey was collected directly from the cohort children themselves as they grew older, it is information from parents that is analysed here. The number of families who have been interviewed at least once is 19,243, including 692 families in England who were not recruited until MCS2. If these cases are counted, the initial response rate was 71%. In this study, the sample included one child per family, excluding children who were the second or third in sets of twins and triplets. Group-based trajectories were based on 17,206 children with parent ratings of conduct problems in at least two surveys. The MCS survey team has developed attrition weights to correct for biases due to non-response, alongside the sample weights which take into account the complex sample design (Hansen 2014, 18–22).

### Measures

#### Conduct problems

Conduct problems at ages 3, 5, 7, 11 and 14 years were assessed by the Strengths and Difficulties Questionnaire (SDQ) (Goodman 1997, 2001), completed by the parent, mainly mothers (more than 95%). The SDQ is a screening questionnaire with extensive psychometric support (www.sdqinfo.com). In the MCS, construct, convergent, discriminant, and predictive validity have been established for the SDQ subscales at ages 3, 5 and 7 years, showing good internal reliability with alphas ranging from .66 to .82 for all subscales and .77 to .82 for conduct problems (Croft et al. 2015). At ages 11 and 14 years, the internal reliabilities of conduct problems are acceptable, with alphas of .62 and .64, respectively. The questionnaire assesses conduct problems in the past 6 months using five items: (1) often has temper tantrums or hot tempers; (2) generally obedient, usually does what adults request; (3) often fights with other children or bullies them; (4) often lies or cheats; and (5) steals from home, school or elsewhere. To ensure that levels of conduct problems are clinically meaningful, SDQ bandings were based on externally given UK norms at each age (Meltzer et al. 2000), where 10% in that reference sample with the highest scores were considered to be at high risk of conduct problems (0 = not high risk; 1 = high risk). In this sample, 10% of the children were considered to be high risk of conduct problems (*SD* = 0.30) from ages 5 to 14 years, with 20% (*SD* = 0.40) at age 3 years. The higher rate at age 3 years may reflect the lack of established age norms for 3 year-olds (see Meltzer et al. 2000).

#### Time-invariant covariates

Table 1 presents non-time-varying covariates, including gender (1 = male; 0 = female), race/ethnicity (1 = Ethnic Minority; 0 = White British), teenage mother (1 = mother aged 19 years or younger at child’s birth; 0 = mother aged 20 years or older at child’s birth), smoked during pregnancy with child (1 = mother smoked; 0 = no smoking) and low birth weight (1 = less than 2.5 kg; 0 = other).

**Table 1:** Means and standard deviations (SD) of covariates, individual risk factors and cumulative risk scores by trajectory group

	Trajectory group								
Variable	Low		Adolescent-onset		Childhood-limited		Persistent		
	Mean	SD	Mean	SD	Mean	SD	Mean	SD	*F*-Test
*Covariates*									
Male	0.49^a^	(0.49)	0.54^b^	(0.51)	0.54^b^	(0.51)	0.63^c^	(0.50)	F(3,16,534) = 29.32^***^
Ethnic minority	0.10^a^	(0.30)	0.10^a^	(0.30)	0.12^a^	(0.33)	0.06^b^	(0.25)	F(3,16,494) = 9.88^***^
Maternal smoking in pregnancy	0.18^a^	(0.38)	0.32^b^	(0.47)	0.32^b^	(0.47)	0.48^c^	(0.52)	F(3,16,532) = 138.81^**^
Teenage mother	0.05^a^	(0.22)	0.10^b^	(0.30)	0.10^b^	(0.30)	0.15^c^	(0.36)	F(3,17,204) = 61.79^***^
Low birth weight	0.06	(0.24)	0.09	(0.29)	0.06	(0.24)	0.09	(0.29)	F(3,17,204) = 5.58^***^
*Child Factors*									
SDQ emotional problems									
Age 3 years	0.04^a^	(0.21)	0.12^b^	(0.33)	0.16^c^	(0.38)	0.20^d^	(0.41)	F(3,14,732) = 204.25^***^
Age 5 years	0.07^a^	(0.24)	0.11^b^	(0.32)	0.17^c^	(0.38)	0.30^d^	(0.48)	F(3,14,719) = 229.10^***^
Age 7 years	0.08^a^	(0.27)	0.16^b^	(0.38)	0.21^b^	(0.42)	0.38^c^	(0.50)	F(3,13,437) = 284.90^***^
Age 11 years	0.07^a^	(0.25)	0.25^b^	(0.44)	0.13^c^	(0.34)	0.36^d^	(0.50)	F(3,12,792) = 320.46^***^
Age 14 years	0.08^a^	(0.27)	0.31^b^	(0.48)	0.16^c^	(0.38)	0.36^d^	(0.49)	F(3,11,320) = 258.15^***^
SDQ peer problems									
Age 3 years	0.08^a^	(0.27)	0.14^b^	(0.35)	0.20^c^	(0.41)	0.23^c^	(0.44)	F(3,14,633) = 136.19^***^
Age 5 years	0.05^a^	(0.21)	0.09^b^	(0.28)	0.12^c^	(0.33)	0.22^d^	(0.43)	F(3,14,708) = 177.21^***^
Age 7 years	0.05^a^	(0.22)	0.13^b^	(0.34)	0.15^b^	(0.36)	0.31^c^	(0.48)	F(3,13,446) = 279.31^***^
Age 11 years	0.07^a^	(0.25)	0.24^b^	(0.44)	0.14^c^	(0.35)	0.36^d^	(0.50)	F(3,12,796) = 310.35^***^
Age 14 years	0.10^a^	(0.29)	0.35^b^	(0.49)	0.17^c^	(0.39)	0.46^d^	(0.51)	F(3,11,325) = 324.23^***^
SDQ hyperactivity									
Age 3 years	0.08^a^	(0.27)	0.20^b^	(0.41)	0.29^c^	(0.47)	0.42^d^	(0.51)	F(3,14,622) = 502.87^***^
Age 5 years	0.05^a^	(0.20)	0.12^b^	(0.33)	0.20^c^	(0.41)	0.47^d^	(0.52)	F(3,14,653) = 736.83^***^
Age 7 years	0.06^a^	(0.24)	0.19^b^	(0.40)	0.22^b^	(0.43)	0.51^c^	(0.52)	F(3,13,416) = 688.69^***^
Age 11 years	0.05^a^	(0.21)	0.29^b^	(0.46)	0.12^c^	(0.34)	0.44^d^	(0.52)	F(3,12,765) = 665.09^***^
Age 14 years	0.04^a^	(0.19)	0.28^b^	(0.47)	0.10^c^	(0.31)	0.42^d^	(0.51)	F(3,11,315) = 637.79^***^
Low verbal cognitive ability									
Age 3 years	0.04^a^	(0.19)	0.07^b^	(0.27)	0.07^b^	(0.27)	0.08^b^	(0.28)	F(3,14,502) = 16.94^***^
Age 5 years	0.04^a^	(0.20)	0.08^b^	(0.27)	0.07^b^	(0.27)	0.09^b^	(0.29)	F(3,14,909) = 22.01^***^
Age 7 years	0.03^a^	(0.17)	0.09^b^	(0.27)	0.08^b^	(0.27)	0.17^c^	(0.35)	F(3,13,386) = 127.36^***^
Age 11 years	0.04^a^	(0.19)	0.09^b^	(0.29)	0.07^b^	(0.37)	0.14^c^	(0.36)	F(3,12,968) = 64.62^***^
Age 14 years	0.05^a^	(0.21)	0.10^b^	(0.31)	0.08^b^	(0.28)	0.12^c^	(0.34)	F(3,10,768) = 33.95^***^
*Family Factors*									
Non-intact families									
Age 9 months	0.11^a^	(0.31)	0.17^b^	(0.38)	0.19^b^	(0.40)	0.29^c^	(0.47)	F(3,16,532) = 105.67^***^
Age 3 years	0.14^a^	(0.34)	0.26^b^	(0.45)	0.26^b^	(0.45)	0.39^c^	(0.50)	F(3,15,470) = 170.80^***^
Age 5 years	0.19^a^	(0.39)	0.33^b^	(0.48)	0.30^b^	(0.47)	0.42^c^	(0.51)	F(3,15,184) = 145.39^***^
Age 7 years	0.21^a^	(0.40)	0.36^b^	(0.49)	0.34^b^	(0.49)	0.47^c^	(0.52)	F(3,13,833) = 142.74^***^
Age 11 years	0.28^a^	(0.44)	0.47^b^	(0.51)	0.39^c^	(0.50)	0.58^d^	(0.51)	F(3,13,263) = 151.29^***^
Age 14 years	0.30^a^	(0.45)	0.51^b^	(0.51)	0.40^c^	(0.50)	0.61^d^	(0.51)	F(3,11,701) = 131.52^***^
Large family (>3 children)									
Age 9 months	0.20^a^	(0.40)	0.23^a,b^	(0.43)	0.24^b^	(0.44)	0.26^b^	(0.45)	F(3,16,532) = 9.86^***^
Age 3 years	0.26^a^	(0.43)	0.30^b^	(0.47)	0.32^b^	(0.48)	0.31^b^	(0.48)	F(3,15,473) = 11.96^***^
Age 5 years	0.33^a^	(0.46)	0.38^b^	(0.49)	0.39^b^	(0.50)	0.43^b^	(0.51)	F(3,15,184) = 18.18^***^
Age 7 years	0.38^a^	(0.48)	0.44^b^	(0.51)	0.44^b^	(0.51)	0.51^b^	(0.52)	F(3,13,832) = 23.13^***^
Age 11 years	0.39^a^	(0.48)	0.48^b^	(0.51)	0.46^b^	(0.51)	0.53^b^	(0.52)	F(3,13,260) = 27.60^***^
Age 14 years	0.36^a^	(0.47)	0.46^b^	(0.51)	0.42^b^	(0.51)	0.50^b^	(0.51)	F(3,11,701) = 22.30^***^
Parental depressive symptoms									
Age 3 years	0.02^a^	(0.15)	0.07^b^	(0.26)	0.07^b^	(0.26)	0.12^c^	(0.34)	F(3,14,435) = 115.31^***^
Age 5 years	0.03^a^	(0.16)	0.08^b^	(0.27)	0.07^b^	(0.27)	0.13^c^	0(.35)	F(3,14,570) = 109.77^***^
Age 7 years	0.03^a^	(0.16)	0.08^b^	(0.29)	0.07^b^	(0.27)	0.14^c^	(0.36)	F(3,13,361) = 107.46^***^
Age 11 years	0.01^a^	(0.12)	0.06^b^	(0.24)	0.05^b^	(0.22)	0.10^c^	(0.32)	F(3,12,675) = 83.29^***^
Age 14 years	0.01^a^	(0.12)	0.05^b,c^	(0.23)	0.04^b^	(0.21)	0.08^c^	(0.28)	F(3,11,109) = 40.82^***^
Parental poor health									
Age 9 months	0.03^a^	(0.17)	0.07^b^	(0.26)	0.06^b^	(0.24)	0.09^b^	(0.29)	F(3,16,526) = 37.60^***^
Age 3 years	0.03^a^	(0.16)	0.06^b^	(0.24)	0.06^b^	(0.24)	0.09^c^	(0.30)	F(3,15,418) = 40.23^***^
Age 5 years	0.03^a^	(0.17)	0.08^c^	(0.27)	0.06^b^	(0.24)	0.08^c^	(0.28)	F(3,15,138) = 31.48^***^
Age 7 years	0.03^a^	(0.17)	0.07^b^	(0.26)	0.06^b^	(0.24)	0.08^b^	(0.28)	F(3,13,782) = 23.30^***^
Age 11 years	0.02^a^	(0.14)	0.08^b^	(0.27)	0.06^b^	(0.24)	0.12^c^	(0.34)	F(3,13,197) = 24.23^***^
Age 14 years	0.02^a^	(0.14)	0.06^b^	(0.25)	0.05^b^	(0.22)	0.09^b^	(0.30)	F(3,11,959) = 43.99^***^
*Socio-economic Factors*									
Lowest income quintile									
Age 9 months	0.14^a^	(0.34)	0.27^b^	(0.45)	0.25^b^	(0.45)	0.39^c^	(0.50)	F(3,16,477) = 187.54^***^
Age 3 years	0.13^a^	(0.33)	0.23^b^	(0.43)	0.27^b^	(0.46)	0.38^c^	(0.50)	F(3,15,322) = 208.35^***^
Age 5 years	0.14^a^	(0.34)	0.25^b^	(0.44)	0.24^b^	(0.44)	0.36^c^	(0.50)	F(3,15,090) = 136.95^***^
Age 7 years	0.12^a^	(0.32)	0.22^b^	(0.42)	0.23^b^	(0.43)	0.32^c^	(0.48)	F(3,13,815) = 122.20^***^
Age 11 years	0.09^a^	(0.22)	0.22^b^	(0.43)	0.19^b^	(0.40)	0.33^c^	(0.49)	F(3,13,260) = 185.08^***^
Age 14 years	0.07^a^	(0.26)	0.20^b^	(0.41)	0.18^b^	(0.40)	0.31^c^	(0.48)	F(3,11,698) = 160.79^***^
Worklessness									
Age 9 months	0.12^a^	(0.32)	0.25^b^	(0.44)	0.23^b^	(0.43)	0.35^c^	(0.50)	F(3,16,529) = 197.34^***^
Age 3 years	0.10^a^	(0.30)	0.21^b^	(0.42)	0.24^b^	(0.44)	0.37^c^	(0.50)	F(3,15,384) = 253.23^***^
Age 5 years	0.11^a^	(0.31)	0.21^b^	(0.41)	0.21^b^	(0.42)	0.35^c^	(0.49)	F(3,15,180) = 184.86^***^
Age 7 years	0.09^a^	(0.28)	0.21^b^	(0.42)	0.19^b^	(0.41)	0.34^c^	(0.49)	F(3,13,830) = 199.20^***^
Age 11 years	0.09^a^	(0.28)	0.22^b^	(0.42)	0.17^c^	(0.39)	0.35^d^	(0.49)	F(3,13,260) = 186.23^***^
Age 14 years	0.07^a^	(0.25)	0.18^b^	(0.40)	0.13^c^	(0.35)	0.31^d^	(0.48)	F(3,11,698) = 180.68^***^
Low qualifications									
Age 9 months	0.15^a^	(0.35)	0.27^b.c^	(0.44)	0.23^b^	(0.43)	0.29^c^	(0.47)	F(3,14,802) = 73.67^***^
Age 3 years	0.14^a^	(0.35)	0.27^b,c^	(0.45)	0.22^b^	(0.43)	0.28^c^	(0.47)	F(3,14,097) = 72.86^***^
Age 5 years	0.14^a^	(0.34)	0.27^b,c^	(0.44)	0.22^b^	(0.42)	0.28^c^	(0.46)	F(3,13,895) = 66.84^***^
Age 7 years	0.13^a^	(0.33)	0.24^b,c^	(0.43)	0.20^b^	(0.41)	0.27^c^	(0.46)	F(3,12,820) = 65.62^***^
Age 11 years	0.12^a^	(0.32)	0.22^b,c^	(0.42)	0.19^c^	(0.41)	0.24^b^	(0.44)	F(3,12,353) = 55.44^***^
Age 14 years	0.11^a^	(0.31)	0.21^b,c^	(0.41)	0.18^b^	(0.40)	0.20^c^	(0.41)	F(3,11,003) = 42.42^***^
Social housing									
Age 9 months	0.17^a^	(0.37)	0.33^b^	(0.48)	0.30^b^	(0.47)	0.46^c^	(0.51)	F(3,16,492) = 202.30^***^
Age 3 years	0.16^a^	(0.36)	0.31^b^	(0.46)	0.32^b^	(0.46)	0.47^c^	(0.51)	F(3,15,366) = 243.54^***^
Age 5 years	0.16^a^	(0.36)	0.31^b^	(0.47)	0.29^b^	(0.47)	0.46^c^	(0.51)	F(3,15,109) = 211.20^***^
Age 7 years	0.14^a^	(0.35)	0.28^b^	(0.46)	0.29^b^	(0.47)	0.45^c^	(0.51)	F(3,13,703) = 197.10^***^
Age 11 years	0.14^a^	(0.34)	0.29^b^	(0.47)	0.27^b^	(0.46)	0.42^c^	(0.51)	F(3,13,304) = 184.66^***^
Age 14 years	0.12^a^	(0.33)	0.27^b^	(0.46)	0.25^b^	(0.45)	0.39^c^	(0.50)	F(3,11,466) = 145.50^***^
*Cumulative Risk Scores*									
Child risk									
Age 3 years	0.24^a^	(0.52)	0.51^b^	(0.78)	0.68^c^	(0.86)	0.90^d^	(0.96)	F(3,13,681) = 534.32^***^
Age 5 years	0.19^a^	(0.47)	0.39^b^	(0.69)	0.53^c^	(0.80)	1.04^d^	(1.01)	F(3,14,377) = 698.81^***^
Age 7 years	0.22^a^	(0.53)	0.55^b^	(0.81)	0.62^b^	(0.93)	1.34^c^	(1.04)	F(3,12,969) = 900.07^***^
Age 11 years	0.21^a^	(0.53)	0.82^b^	(1.02)	0.44^c^	(0.77)	1.30^d^	(1.15)	F(3,12,448) = 839.95^***^
Age 14 years	0.26^a^	(0.56)	1.01^b^	(1.07)	0.50^c^	(0.80)	1.31^d^	(1.13)	F(310,467) = 654.68^***^
Family risk									
Age 9 months	0.34^a^	(0.53)	0.47^b^	(0.62)	0.49^b^	(0.64)	0.64^c^	(0.72)	F(3,16,526) = 112.35^***^
Age 3 years	0.43^a^	(0.60)	0.67^b^	(0.73)	0.68^b^	(0.75)	0.89^c^	(0.88)	F(3,13,578) = 202.12^***^
Age 5 years	0.56^a^	(0.65)	0.85^b^	(0.80)	0.81^b^	(0.80)	1.06^c^	(0.90)	F(3,14,325) = 218.04^***^
Age 7 years	0.64^a^	(0.68)	0.95^b^	(0.83)	0.90^b^	(0.82)	1.20^c^	(0.88)	F(3,13,157) = 214.77^***^
Age 11 years	0.72^a^	(0.71)	1.09^b^	(0.81)	0.97^c^	(0.80)	1.32^d^	(0.91)	F(3,12,369) = 216.74^***^
Age 14 years	0.71^a^	(0.72)	1.12^b^	(0.85)	0.94^c^	(0.84)	1.32^d^	(0.92)	F(3,10,911) = 180.46^***^
Socio-economic risk									
Age 9 months	0.50^a^	(0.89)	1.01^b^	(1.22)	0.87^c^	(1.18)	1.27^d^	(1.31)	F(3,14,772) = 243.22^***^
Age 3 years	0.47^a^	(0.86)	0.92^b^	(1.19)	0.92^b^	(1.23)	1.28^c^	(1.33)	F(3,13,913) = 289.17^***^
Age 5 years	0.48^a^	(0.87)	0.95^b^	(1.18)	0.83^b^	(1.16)	1.25^c^	(1.31)	F(3,13,824) = 234.37^***^
Age 7 years	0.43^a^	(0.81)	0.87^b^	(1.15)	0.81^b^	(1.15)	1.21^c^	(1.27)	F(3,12,710) = 240.68^***^
Age 11 years	0.38^a^	(0.79)	0.88^b^	(1.17)	0.73^c^	(1.10)	1.19^d^	(1.33)	F(3,12,160) = 244.34^***^
Age 14 years	0.34^a^	(0.71)	0.78^b^	(1.04)	0.66^c^	(1.06)	1.06^d^	(1.28)	F(3,10,816) = 211.48^***^

Post-hoc analyses using Bonferroni’s method identified significant pairwise comparisons (*p* < 0.05) between groups, shown when group means do not share any similar superscripts

*p< 0.05, **p< 0.001, ***p < 0.001

#### Child risk factors

##### Low verbal ability

The British Ability Scale test scores were used; at ages 3 and 5 years, the Naming Vocabulary test score; at age 7 years, the Word Reading test score; at age 11 years, the Verbal similarity test score; and at age 14 years, the Word Activity test scores, which are subsets of those used in a vocabulary assessment in the 1970 British Cohort Study (BCS70) Age 16 survey. Scores were standardized, those which were 1 SD below the mean were considered low.

##### Co-morbid problems

The SDQ (Goodman 1997, 2001) was used to assess (i) inattention/hyperactivity (alphas = 0.79 and 0.78 at ages 11 and 14 years, respectively) with five items such as easily distracted, concentration wanders and thinks things out before acting (reversed); (ii) peer problems (alphas = 0.64 and 0.63 at ages 11 and 14 years, respectively) with five items including has at least one good friend (reversed) and rather solitary, tends to play alone; and (iii) emotional problems (alphas = 0.71 and 0.73 at ages 11 and 14 years, respectively) with five items including many fears, easily scared and many worries, often seems worried. In all cases, each item is marked according to 0 = not true, 1 = somewhat true and 2 = very true, and SDQ bandings were used, where the 10% of children in the national reference sample with the highest scores in each symptom group were considered to be at high risk (0 = not high risk; 1 = high risk).

#### Family risk factors

##### Non-intact families

Families where the two biological parents did not live together were considered to be a risk factor, (1 = single and step-parent families; 0 = two biological parents).

##### Large family size

Families with three or more children were considered to be large and treated as a risk factor because of possible competition for parental attention and family resources (De La Rochebrochard and Joshi 2013). Three children are equivalent to one standard deviation above the mean for number of children per family and also represent deviation from the “two-child norm”, (1 = 3 or more children; 0 = 2 or less children).

##### Parental depressive symptoms (alphas range from .88 to .90)

Mothers and fathers answered 6 items from the Kessler Scale (Kessler et al. 2002) including, “During the last 30 days, about how often did you feel hopeless?” and “During the last 30 days, about how often did you feel that everything was an effort?” If either parent had a score of 13 or more, the risk score was equal to 1. The cut-point of 13 was developed to operationalize the definition of serious mental illness, defined as meeting diagnostic criteria for a DSM-IV disorder in the past 12-months, and has been shown to be clinically valid in population-based surveys (Kessler et al. 2002).

##### Poor parental physical health

Mothers and fathers were asked if they had a longstanding physical health conditions. If either parent answered yes, the risk score was equal to 1.

#### Socio-economic risk factors

##### Low parental education

If neither the mother nor father had any formal qualifications from school or college, the risk factor was 1; otherwise 0.

##### Low household income

A measure of net family income was derived for general use by the survey team (see Ketende and Joshi 2008 for details). This had imputed missing values, adjusted for family size (equivalized), and for survey weighting, dividing families into five equally sized income bands (quintiles). The lowest quintile was treated as the risk factor (1 = lowest quintile; 0 = any other quintile).

##### Parental worklessness

Households with no employed parent were considered at risk (1 = worklessness; 0 = at least one parent employed).

##### Social housing

Families currently renting from local authorities or housing associations were treated at risk (1 = living in social housing; 0 = not living in social housing).

### Statistical Analyses

Group-based trajectory analysis in STATA traj (Jones and Nagin 2013) was used to model trajectories as a function of each child’s age measured in months at each interview. Group-based trajectory modeling is a specialized form of finite mixture modeling (see Nagin 2005; Nagin and Odgers; 2010). Trajectories were modelled with only observed data and sampling weights reflecting the MCS design were applied. Full Information Maximum Likelihood (FIML) estimated the model parameters, thereby including every case with at least two maternal ratings (Schafer and Graham 2002). Binary logit distribution was specified as conduct problems are treated as a dichotomous variable (e.g., whether clinically meaningful or not). To establish the best fitting solution a range of fit indicators were examined, including the lowest absolute Bayesian Information Criterion (BIC), the average posterior probability of group membership (.70 to .80 being acceptable), a close correspondence between the estimated probability of group membership, and the proportion assigned to that group based on the posterior probability of group membership (Andruff et al. 2009; Nagin 2005).

In order to account for the complex clustered and stratified survey design of MCS, *svy* in STATA was used, as well as attrition weights to restore the original profile of the whole cohort. First, the proportions and standard deviations of the covariates, the individual risk factors and cumulative risk scores (i.e., child, family and socio-economic) were examined at each age by trajectory group (see Table 1). To do this, regressions were run for each risk factor on trajectory group status and then post-hoc tests were conducted to compare all possible pairwise differences among the four groups, using the Bonferroni correction. Next, the effects of timing and persistence of the cumulative risk scores were assessed. First, the VIF (*variance inflation factor*) command was used to check for collinearity among the cumulative risk scores. The VIF values were lower than 10, indicating that collinearity was not a problem. Then, a series of multinomial logistic regressions were run for the trajectory groups, where the multiple cumulative risk scores for each domain were entered consecutively at each age, controlling for the covariates and previous cumulative risk scores (Table 2). Further testing examined whether there were interactive effects among the cumulative risk scores, using multiplicative terms; but none were significant.

**Table 2:** Multinomial logistic regression predicting trajectory groups

	Adolescent-onset		Childhood-limited		Persistent	
	*RRR*	*CI*	*RRR*	*CI*	*RRR*	*CI*
**Model 1 (*N*** = 14,749)						
*Age 9 months*						
Male	1.29^**^	(1.10, 1.51)	1.26***	(1.13, 1.41)	1.93^***^	(1.61, 2.31)
Ethnic minority	0.78^*^	(0.60, 0.99)	1.07	(0.90, 1.27)	0.54^***^	(0.40, 0.74)
Low birth weight	1.41^*^	(1.04, 1.90)	0.82	(0.64, 1.05)	1.24	(0.91, 1.71)
Teenage mother	1.16	(0.87, 1.55)	1.45^***^	(1.18, 1.79)	1.34^*^	(1.00, 1.81)
Smoking in pregnancy	1.58^***^	(1.30, 1.90)	1.73^***^	(1.52, 1.97)	2.43^***^	(2.00, 2.95)
Family risk	1.03	(0.88, 1.20)	1.12^*^	(1.01, 1.25)	1.21^*^	(1.03, 1.41)
Socio-economic risk	1.49^***^	(1.37, 1.60)	1.26^***^	(1.19, 1.34)	1.59^***^	(1.46, 1.72)
**Model 2 (*N*** = 11,247)						
*Age 9 months*						
Male	1.26^*^	(1.11, 1.52)	1.16^*^	(1.03, 1.33)	1.83^***^	(1.49, 2.30)
Ethnic minority	0.57^**^	(0.38, 0.79)	0.90	(0.70, 1.09)	0.42^***^	(0.27, 0.67)
Low birth weight	1.37	(1.00, 2.06)	0.60^**^	(0.45, 0.87)	0.77	(0.53, 1.30)
Teenage mother	1.12	(0.80, 1.62)	1.50^***^	(1.20, 1.97)	1.24	(0.90, 1.87)
Smoking in pregnancy	1.43^**^	(1.14, 1.80)	1.54^***^	(1.33, 1.80)	2.35^***^	(1.85, 2.99)
Family risk	0.84	(0.68, 1.08)	0.90	(0.77, 1.07)	0.89	(0.70, 1.16)
Socio-economic risk	1.33^***^	(1.18, 1.57)	1.01	(0.94, 1.14)	1.17^*^	(1.01, 1.36)
*Age 3 years*						
Family risk	1.33^**^	(1.12, 1.63)	1.32^***^	(1.16, 1.52)	1.55^***^	(1.25, 1.93)
Socio-economic risk	1.07	(0.94, 1.28)	1.25^***^	(1.16, 1.40)	1.30^***^	(1.13, 1.49)
Child risk	1.88^***^	(1.63, 2.19)	2.47^***^	(2.25, 2.74)	3.08^***^	(2.68, 3.52)
**Model 3 (*N*** = 9,889)						
*Age 9 months*						
Male	1.24^*^	(1.02, 1.50)	1.12	(0.97, 1.28)	1.67^***^	(1.33, 2.14)
Ethnic minority	0.52^**^	(0.34, 0.79)	0.85	(0.63, 1.13)	0.42^***^	(0.22, 0.61)
Low birth weight	1.40	(0.95, 2.06)	0.60^**^	(0.42, 0.85)	0.72	(0.44, 1.16)
Teenage mother	1.18	(0.81, 1.72)	1.55^**^	(1.17, 2.04)	1.11	(0.74, 1.76)
Smoking in pregnancy	1.45^**^	(1.15, 1.83)	1.63^***^	(1.38, 1.93)	2.58^***^	(1.99, 3.35)
Family risk	0.77^*^	(0.60, 0.98)	0.90	(0.75, 1.08)	0.86	(0.67, 1.16)
Socio-economic risk	1.30^**^	(1.10, 1.54)	0.99	(0.88, 1.10)	1.09	(0.95, 1.35)
*Age 3 years*						
Family risk	1.02	(0.80, 1.29)	1.12	(0.94, 1.35)	1.16	(0.90, 1.54)
Socio-economic risk	0.95	(0.79, 1.14)	1.23^**^	(1.08, 1.41)	1.21	(1.00, 1.43)
Child risk	1.70^***^	(1.45, 2.00)	2.03^***^	(1.82, 2.27)	2.17^***^	(1.86, 2.59)
*Age 5 years*						
Family risk	1.54^***^	(1.27, 1.87)	1.21^*^	(1.05, 1.42)	1.40^**^	(1.11, 1.75)
Socio-economic risk	1.14	(0.96, 1.35)	0.98	(0.87, 1.12)	1.11	(0.96, 1.39)
Child risk	1.50^***^	(1.25, 1.80)	2.02^***^	(1.77, 2.30)	3.46^***^	(2.96, 4.06)
**Model 4 (*N*** = 8,537)						
*Age 9 months*						
Male	1.22^*^	(1.00, 1.50)	1.10	(0.94, 1.28)	1.49^**^	(1.15, 1.95)
Ethnic minority	0.50^**^	(0.31, 0.82)	1.00	(0.73, 1.37)	0.49^*^	(0.25, 0.76)
Low birth weight	1.24	(0.81, 1.90)	0.50^***^	(0.33, 0.75)	0.47^*^	(0.28, 0.89)
Teenage mother	1.01	(0.65, 1.57)	1.49^*^	(1.08, 2.04)	1.03	(0.62, 1.73)
Smoking in pregnancy	1.38^**^	(1.07, 1.78)	1.55^***^	(1.29, 1.86)	2.64^***^	(1.97, 3.53)
Family risk	0.72^*^	(0.55, 0.93)	0.90	(0.73, 1.10)	0.72^*^	(0.54, 0.98)
Socio-economic risk	1.37^***^	(1.14, 1.65)	0.94	(0.82, 1.07)	1.11	(0.93, 1.40)
*Age 3 years*						
Family risk	0.97	(0.74, 1.25)	1.10	(0.90, 1.34)	1.22	(0.90, 1.65)
Socio-economic risk	0.86	(0.71, 1.05)	1.23^**^	(1.05, 1.43)	1.14	(0.91, 1.44)
Child risk	1.54^***^	(1.29, 1.83)	1.82^***^	(1.60, 2.06)	1.84^***^	(1.54, 2.24)
*Age 5 years*						
Family risk	1.34^*^	(1.04, 1.74)	1.09	(0.90, 1.33)	1.10	(0.80, 1.49)
Socio-economic risk	1.10	(0.89, 1.35)	0.88	(0.75, 1.03)	0.96	(0.79, 1.32)
Child risk	1.31^**^	(1.07, 1.61)	1.67^***^	(1.44, 1.95)	2.05^***^	(1.68, 2.49)
*Age 7 years*						
Family risk	1.28^*^	(1.02, 1.60)	1.12	(0.95, 1.32)	1.29^*^	(1.01, 1.64)
Socio-economic risk	1.04	(0.86, 1.27)	1.19^*^	(1.03, 1.38)	1.17	(0.95, 1.50)
Child risk	1.56^***^	(1.33, 1.83)	1.69^***^	(1.49, 1.92)	2.88^***^	(2.47, 3.42)
**Model 5 (*N*** = 7,401)						
*Age 9 months*						
Male	1.30^*^	(1.04, 1.61)	1.06	(0.90, 1.25)	1.61^***^	(1.21, 2.15)
Ethnic minority	0.54^*^	(0.32, 0.90)	1.00	(0.70, 1.42)	0.42^**^	(0.22, 0.80)
Low birth weight	1.26	(0.81, 1.96)	0.51^**^	(0.33, 0.79)	0.51^*^	(0.28, 0.92)
Teenage mother	1.03	(0.63, 1.68)	1.63^**^	(1.14, 2.33)	1.11	(0.65, 1.89)
Smoking in pregnancy	1.43^*^	(1.09, 1.87)	1.62^***^	(1.33, 1.98)	2.65^***^	(1.92, 3.65)
Family risk	0.76	(0.58, 1.01)	0.93	(0.74, 1.16)	0.83	(0.59, 1.16)
Socio-economic risk	1.41^***^	(1.16, 1.72)	0.95	(0.82, 1.11)	1.02	(0.82, 1.27)
*Age 3 years*						
Family risk	0.96	(0.72, 1.28)	1.13	(0.90, 1.43)	1.07	(0.76, 1.50)
Socio-economic risk	0.79	(0.64, 0.98)	1.11	(0.93, 1.32)	1.18	(0.92, 1.52)
Child risk	1.49^***^	(1.23, 1.81)	1.93^***^	(1.68, 2.21)	1.75^***^	(1.42, 2.16)
*Age 5 years*						
Family risk	1.26	(0.95, 1.66)	1.00	(0.81, 1.24)	1.01	(0.72, 1.42)
Socio-economic risk	1.17	(0.94, 1.46)	0.94	(0.78, 1.12)	1.11	(0.85, 1.46)
Child risk	1.04	(0.83, 1.30)	1.55^***^	(1.31, 1.84)	1.75^***^	(1.41, 2.18)
*Age 7 years*						
Family risk	1.06	(0.80, 1.40)	1.19	(0.97, 1.47)	1.02	(0.75, 1.39)
Socio-economic risk	0.92	(0.73, 1.18)	1.14	(0.95, 1.38)	0.92	(0.70, 1.20)
Child risk	1.15	(0.95, 1.39)	1.65^***^	(1.43, 1.91)	2.12^***^	(1.75, 2.56)
*Age 11 years*						
Family risk	1.33^*^	(1.06, 1.67)	1.02	(0.86, 1.21)	1.48^**^	(1.14, 1.92)
Socio-economic risk	1.21	(0.97, 1.52)	1.09	(0.93, 1.28)	1.34^*^	(1.06, 1.68)
Child risk	2.33^***^	(2.00, 2.71)	1.14	(0.98, 1.31)	2.18^***^	(1.83, 2.59)
**Model 6 (*N*** = 5,912)						
*Age 9 months*						
Male	1.55^***^	(1.21, 1.99)	1.06	(0.88, 1.28)	1.74^***^	(1.25, 2.41)
Ethnic minority	0.61	(0.35, 1.09)	1.02	(0.69, 1.50)	0.42^*^	(0.19, 0.94)
Low birth weight	1.48	(0.88, 2.49)	0.53^*^	(0.32, 0.86)	0.52	(0.25, 1.08)
Teenage mother	1.08	(0.59, 1.96)	1.51	(0.99, 2.30)	1.14	(0.60, 2.17)
Smoking in pregnancy	1.38^*^	(1.00, 1.90)	1.68^***^	(1.33, 2.12)	2.02^***^	(1.37, 2.98)
Family risk	0.78	(0.57, 1.07)	1.02	(0.78, 1.31)	0.89	(0.61, 1.30)
Socio-economic risk	1.43^*^	(1.13, 1.80)	0.91	(0.77, 1.09)	1.15	(0.89, 1.47)
*Age 3 years*						
Family risk	1.02	(0.74, 1.41)	1.11	(0.85, 1.45)	0.93	(0.63, 1.38)
Socio-economic risk	0.73^*^	(0.56, 0.94)	1.18	(0.96, 1.45)	1.20	(0.89, 1.61)
Child risk	1.44^**^	(1.14, 1.80)	1.93^***^	(1.66, 2.25)	1.68^***^	(1.30, 2.17)
*Age 5 years*						
Family risk	1.17	(0.84, 1.62)	0.98	(0.76, 1.25)	1.01	(0.70, 1.47)
Socio-economic risk	1.12	(0.86, 1.45)	0.88	(0.71, 1.09)	1.08	(0.78, 1.49)
Child risk	0.93	(0.72, 1.22)	1.55^***^	(1.28, 1.87)	1.72^***^	(1.33, 2.21)
*Age 7 years*						
Family risk	0.96	(0.69, 1.34)	1.24	(0.97, 1.58)	1.02	(0.72, 1.44)
Socio-economic risk	1.04	(0.78, 1.37)	1.11	(0.90, 1.36)	0.94	(0.68, 1.28)
Child risk	0.96	(0.77, 1.20)	1.49^***^	(1.26, 1.78)	1.90^***^	(1.51, 2.40)
*Age 11 years*						
Family risk	1.28	(0.92, 1.79)	0.92	(0.72, 1.18)	1.25	(0.86, 1.82)
Socio-economic risk	1.07	(0.79, 1.44)	0.99	(0.78, 1.25)	1.16	(0.82, 1.63)
Child risk	1.64^***^	(1.34, 2.01)	1.15	(0.97, 1.37)	1.87^***^	(1.49, 2.34)
*Age 14 years*						
Family risk	1.15	(0.87, 1.52)	1.06	(0.85, 1.33)	1.32	(0.95, 1.84)
Socio-economic risk	1.13	(0.85, 1.50)	1.24	(0.99, 1.57)	1.00	(0.70, 1.39)
Child risk	2.36^***^	(1.99, 2.80)	1.13	(0.96, 1.32)	1.82^***^	(1.48, 2.24)

*p < 0.05, ** *p* < 0.01, ***p < 0.001

## Results

### Trajectories of Conduct Problems

Using group-based trajectory modeling, models with three to five trajectories with linear to cubic functional forms were tested. The four-group, cubic model fit the data best. The BIC score for the four group, cubic model (−21584.54) had the absolute lowest score compared to the three (−21617.30) and five (−21586.76) group, cubic models, and the three (−21623.23), four (−21602.63) and five (−21601.46) group, quadratic models. For the four-group, cubic model, the mean posterior probability scores, indexing the degree to which the data fit the assigned trajectory, ranged from 0.76 to 0.79, with a mean of 0.77, indicating a satisfactory fit. Furthermore, estimated proportions of the four-group, cubic model were similar to actual proportions of each group. The four-group, quadratic model revealed similar trajectories as the four-group, cubic model, but had slightly lower mean posterior probability scores. The five group, quadratic and cubic models revealed the same four trajectories, with an additional moderate, stable pathway. The four-group, cubic model was chosen, based on the fit indicators and in coherence with previous studies.

**Fig. 1:**
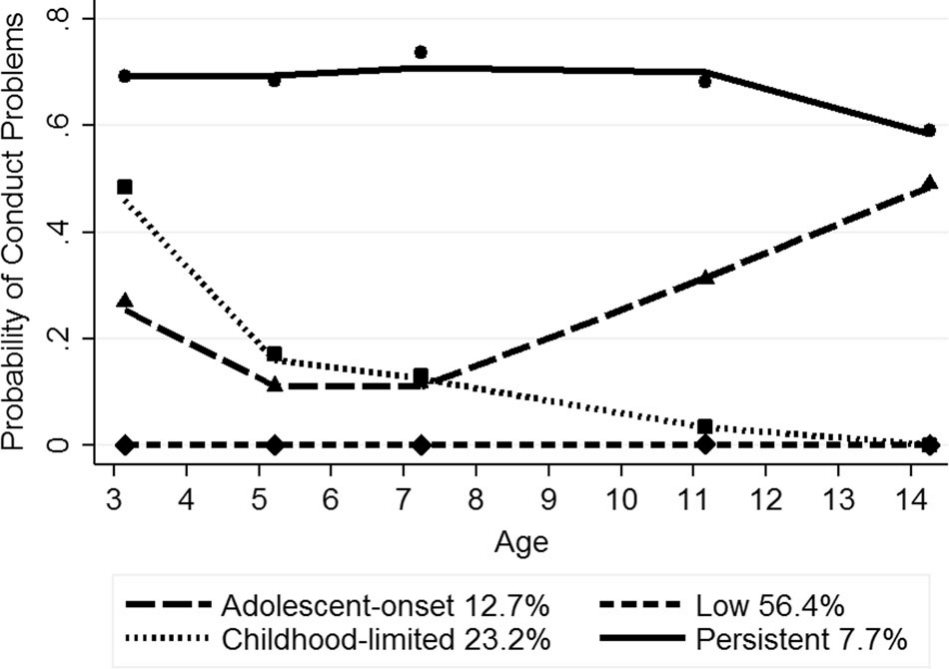
Trajectory groups of conduct problems from ages 3 to 14 years. Shown are estimated trajectories (lines), observed group means at each age (markers) and estimated group percentages

Figure 1 depicts the probability of clinically relevant conduct problems for the four trajectory groups from ages 3 to 14 years, along with the estimated weighted proportion in each group (total N of observation occasions = 67,090). As shown in Fig. 1, the predicted and observed means were close, indicating a good fit of the model. Furthermore, estimated and actual proportions of each group were similar. The groups identified include one low problem pathway and three conduct problem pathways, labeled as persistent, childhood-limited and adolescent-onset. The low group (56.4% estimated; 57.9% actual) showed a stable trajectory of a low probability of conduct problems from ages 3 to 14 years. The childhood-limited group (23.2% estimated; 22.6% actual) demonstrated a moderate probability of conduct problems in early childhood (around 50%) with a decline from ages 3 to 5 years and a low, stable probability of conduct problems thereafter. The persistent group (7.7% estimated; 7.2% actual) followed a very high probability of continuous high level conduct problems, which declined slightly from age 11 years but was still close to 60% at age 14 years. The adolescent-onset group (12.7% estimated; 12.4% actual) displayed a moderate probability of conduct problems from ages 3 and 7 years, with a substantial increase by age 11 years, rising to around 50% at age 14 years.

### Covariates and Individual Risk Factors

Table 1 presents the proportions and SD of the covariates and individual risk factors at every age by trajectory group. Superscripts reflect post-hoc analyses, where all possible pairwise differences among the four groups were compared. Significant differences were found for those groups who do not share any particular superscripts. There was a higher prevalence of boys in the three conduct problem groups whereas girls were more prevalent in the low group. For ethnic minority children, there was a slightly lower proportion in the persistent group compared to the other groups. There were more children of teenage mothers and mothers who smoked in pregnancy in the three conduct problem groups compared to the low group, with the persistent group having the highest prevalence.

For the individual and cumulative risk factors, the low trajectory showed consistently the lowest levels (with the exception of low birth weight) compared to the three conduct problem pathways, with the persistent group mostly having the highest levels. Compared to the childhood-limited group, the adolescent-onset group had similar or lower levels of co-morbid problems and cumulative child risk in the early years but showed a higher prevalence at ages 11 and 14 years. Other individual and cumulative risk factors for the childhood-limited and adolescent-onset groups were mostly at the intermediate level and not significantly different from one another, especially in the early years. However, the adolescent-onset group had significantly higher rates of growing up in a single or step-parent family and parental worklessness as well as higher scores on cumulative family and socio-economic risks compared to the childhood-limited group at ages 11 and 14 years.

### Cumulative Risk

The multinomial, multivariate estimates contrast the chances of being in one of the three conduct problem trajectories relative to the low group, controlling for all other variables included in each model. Table 2 shows three sets of relative risk ratios (RRR) and their 95% confidence intervals. Since the variables have been defined in a direction likely to raise risks, estimates or RRR greater than 1 are highlighted, with lower bounds on their confidence intervals also over this threshold. At age 9 months (model 1), socio-economic risk significantly raised the chances of being on the conduct problem pathways compared to the low trajectory, as did exposure to family risk (over and above the time-invariant risks) for the childhood-limited and persistent groups. Having a mother who smoked during pregnancy was a risk factor for all three conduct problem trajectories, having a teenage mother was a risk factor for the persistent and childhood-limited pathways, and low birth weight was a risk factor for the adolescent-onset group. Being White British was a risk factor for the adolescent-onset and persistent groups and being male was a risk factor in predicting any of these trajectories.

Adding cumulative risk at age 3 years (model 2), concurrent socio-economic, family and child risks were significantly associated with all three conduct problem pathways over and above previous risk experiences, with the exception of socio-economic risk for the adolescent-onset pattern. Socio-economic risk measured at age 9 months remained a significant predictor of the adolescent-onset and persistent pathways.

Adding cumulative risk at age 5 years (model 3), concurrent family and child risk were significant factors for all three conduct problem groups. Child risk experienced at age 3 years also remained significant for all three groups. Socio-economic risk continued to be significant for the adolescent-onset group at age 9 months and for the childhood-limited group at age 3 years. Socio-economic risk was no longer a significant predictor for the persistent pathway.

Adding cumulative risk at age 7 years (model 4), concurrent family risk was significant for the adolescent-onset and persistent groups, but was no longer significant for the childhood-limited pathway. Socio-economic risk at age 9 months and family risk at age 5 years continued to be significant for the adolescent-onset group. Concurrent and earlier (at age 3) socio-economic risk were significant for the childhood-limited group. Both concurrent and earlier child risk continued to be significant for all three conduct problem groups.

Adding cumulative risk at age 11 years (model 5), concurrent child and family risks were associated with the adolescent-onset and persistent pathways, while concurrent socio-economic risk was significant for the persistent trajectory only. The effects of previous family risk were no longer significant for any of the groups. For earlier child risk, significant effects were shown at ages 3, 5 and 7 years for the childhood-limited and persistent pathways and at age 3 years for the adolescent-onset trajectory. Socio-economic risk at age 9 months remained significant for the adolescent-onset group.

Adding cumulative risk at age 14 years (model 6), concurrent child risk significantly increased the risk of being on the adolescent-onset and persistent pathways. As before, earlier child risk measured at ages 3, 5 and 7 years was significant for the childhood-limited group and at ages 3 and 11 years for adolescent-onset group. The significant effects of child risk continued at all ages for the persistent group. Family and socio-economic risks were no longer significant except that the adolescent-onset path was associated with higher socio-economic risk measured at age 9 months. Being male remained significant for the adolescent-onset and persistent trajectories and being White British remained significant for the persistent pathway. Maternal smoking in pregnancy continued to be a significant predictor of all three conduct problem trajectories.

## Discussion

Previous studies have identified diverse developmental trajectories of conduct problems and examined their associated risk factors. However, more information regarding the timing and persistence of cumulative risk in shaping variations in conduct problem trajectories is needed for intervention purposes. Using evidence from a current, nationally representative UK cohort study, following the lives of over 17,000 children born in 2000/2, this study identified four developmental trajectories of conduct problems from ages 3 to 14 years; one low problem group and three high problem groups including persistent, childhood-limited and adolescent-onset pathways. Confirming previous evidence (Frick 2012; Moffitt 2006), the three problem trajectories were exposed to high levels of socio-economic disadvantage and family risks from infancy and high levels of child risks during early childhood. Adding to previous research, the findings suggest that high levels of family and child risks continued for those on the persistent and adolescent-onset trajectories; but receded for those on the childhood-limited trajectory. The effects of early socio-economic disadvantage persisted for those on the adolescent-onset trajectory, highlighting the importance of early markers for this later onset group. Maternal smoking in pregnancy continued to be a significant predictor for all three conduct problem groups, up to age 14 years. The findings highlight the crucial role of timing and persistence of exposure to risk, providing evidence for the assumption of cumulative, emerging, latency and maturation effects associated with different trajectory patterns, as well as potentially sensitive periods of developmental adjustment.

### Conduct Problem Trajectories

This study identified similar patterns of conduct problem trajectories as established in previous studies using international community samples (e.g., Broidy et al. 2003), differentiating between a low problem group (58%) and persistent (7%), childhood-limited (23%) and adolescent-onset pathways (12%). There appear, however, to be slight variations in the prevalence of the trajectories based on the time frame of observation. For example, the prevalence of the different trajectory groups is similar to those found in New Zealand (Odgers et al., 2007) and the UK (Barker and Maughan 2009) among children followed from ages 4 to 15 years. Among children followed from ages 4 to 17 years (Sentse et al., 2017), however, the prevalence of the group with persistent conduct problems was reported to be higher (25%). This might be due to differences in the measure of conduct problems, as Sentse et al. (2017) did not use a cut-off score as only a few individuals ranked in the clinical range in their sample, unlike in Barker and Maughan (2009) and this study. Nonetheless, future studies should examine in more detail if indeed the prevalence of persisting conduct problems varies with the time period under observation – or with the operationalization of conduct problems. Furthermore, the adolescent-onset group showed a similar proportion, as well as pattern and shape of the trajectory, as other studies (Barker and Maughan 2009; Sentse et al. 2017). Similar to this study, these studies found an increase in conduct problems by ages 7 or 8 years, rising continuously after that, approximating levels near the persistent group around ages 13 to 14 years. The relative low prevalence of adolescent-onset conduct problems, comprising about 1 in 10 of children, does not support the notion of this group being normative. The normative group is the one with persistently low levels of conduct problems, highlighting the need to pay attention both to those who show severe conduct problems from early childhood, as well as those whose problems emerge in adolescence.

### Individual Risk Factors of Conduct Problem Trajectories

A number of distinct risk factors were identified that seem to implicate different developmental processes. Consistent with previous findings (Frick 2012; Moffitt 2006), children on the persistently high problem trajectory were overwhelmingly White British boys and most likely to experience a number of early risk factors including maternal smoking in pregnancy and a teenage mother, although those in this group who had a teenage mother were a minority (15%). The persistent group also showed the highest levels of co-morbid child-level problems compared to those on the other tracks and the lowest verbal abilities from age 7 years, pointing to cumulative effects (Schoon et al. 2003; Masten and Cicchetti 2010). Although they share many of the same family risk factors as the other conduct problem groups, those in the persistent group were more likely to grow up in single or step-parent families, have a depressed parent and experience socio-economic risk (with the exception of parental low qualifications), emphasizing the critical role of socio-economic adversity as a distinguishing risk domain for this group.

In comparison to the low group, the childhood-limited group continued to have moderate levels of co-morbid child-level problems up to age 14 years, supporting previous research that this group might struggle with other mental health problems (Sentse et al. 2017). Children on the childhood-limited pathway also started with higher levels of co-morbid child-level problems than the adolescent-onset group, but showed similar or lower levels from age 7 years, providing evidence for the assumption of maturation effects (Barker and Maughan 2009; Moffitt 1993; Odgers et al. 2008; Sentse et al. 2017).

Conversely, the adolescent-onset group showed higher co-morbid problems from ages 11 and 14 years than the childhood-limited group, pointing to potential trigger effects of co-occurring child and family risks (Schoon et al. 2003). In terms of family and socio-economic risks, the childhood-limited and adolescent-onset groups experienced similar levels in the earlier years, but those on the adolescent-onset pathway had higher levels of some risk factors at ages 11 and 14 years, such as living in single or step-parent families and parental worklessness. For the adolescent-onset group, the findings thus suggest emerging psychopathology as well as possible trigger effects of parental separation and/or unemployment (Cicchetti and Toth 2009; Rutter and Sroufe 2000).

### Timing and Persistence of Cumulative Risk

Considering the results of the multivariate analysis, the three problem trajectories were exposed to higher levels of socio-economic disadvantage and family risks from early childhood and higher levels of child risks throughout childhood than those on the low problem trajectory, confirming previous evidence (Frick 2012; Moffitt 2006). Higher cumulative family risk was shown at age 9 months for both persistent and childhood-limited trajectories and at ages 3 and 5 years for the three conduct problems pathways. For those on the childhood-limited track, exposure to concurrent family risk receded after age 5 years, while for those on the persistent and adolescent-onset trajectories, exposure continued until age 11 years. In support of the cumulative risk model (Hertzman 1999; Schoon et al. 2003), these findings indicate that differences among the trajectory groups may not only lie in the degree and severity of risk, but also in the persistence of risk factors throughout childhood and adolescence. Results thus illustrate the changing role of the impact of family risk factors in shaping variations in trajectories of conduct problems throughout development.

For child risk, the effects of early risk appeared to persist over time, highlighting the enduring impact of co-morbid problems and low verbal ability on conduct problems. For those on the persistent and adolescent-onset pathways, the findings indicated persistent child risk effects from ages 3 to 14 years, with early child risk continuing to show significance up to age 14, showing the pervasiveness of proximal risk factors. In support of the maturation hypothesis, the importance of concurrent child risk receded after age 7 years for those on the childhood-limited trajectory, while earlier child risk continued to show significance. Thus, child risk showed a similar pattern as family risk for those on the childhood-limited pathway, with lessening effects as this group matures into adolescence.

The assumption of emerging psychopathology was supported by evidence in the adolescent-onset group, which experienced a sharp increase in conduct problems during adolescence, i.e., at ages 11 and 14 years. Potential latency effects, i.e. the fact that risks might not have an immediate but a delayed impact on children’s adjustment (Keiley et al. 2000; Schoon et al. 2003) were evident in the long reach of exposure to early socio-economic disadvantage that affected those on the adolescent-onset trajectory. Together, this suggests that while the emergence of severe conduct problems in early adolescence may differentiate this pathway, there also appears to be early risk markers which make this group more vulnerable to arising problems later. Thus, emerging problems have to be understood in the context of longer-term risk profiles and experiences. While early intervention might be beneficial for this group, the findings also point to the importance of later school-age interventions to support their transition into adolescence and beyond. This emergent trajectory pattern coincides with the transition from primary to secondary school, highlighting this transition period as a particular sensitive and critical period in the lives of young people.

The findings exemplify the role of timing and persistence of risk effects in shaping variations in conduct problem trajectories and provide evidence for the assumption of differential risk effects. While cumulative risk is particularly relevant for the persistent trajectory, maturation effects are mainly associated with the childhood-limited trajectory. The adolescent-onset trajectory is associated with emerging as well as latency risk effects. A better understanding of the changing dynamics of risk effects clearly has important implications for the design of effective interventions, and future research should assess the association between distinct risk effects and variations in conduct problems trajectories in more detail.

### Limitations

In interpreting the findings, a number of limitations should be considered. First, conduct problems and the risk factors were assessed using parental reports. This raises the problem of informant and methodical biases and caution is needed when inferring the meaning and generalizability of the results. Some other studies have relied on teacher- or self-reports rather than parent-reports. It is arguable that teachers may have a more limited familiarity with a child’s development than parents, and in any case, children and teachers cannot provide reports before school-age, as reported in this study. Second, group-based trajectory analysis only provides a descriptive summary of a potential underlying typology in pathways over a defined period of observation. The fit indicators provide some guidelines about the number of types to select and the final selection is based on consideration of parsimony, the BIC statistics and average posterior probability of group membership. Even though four distinct groups of patterns were identified for each gender; the composition, shape and membership of these patterns might change by taking into account later periods of observation. Third, there was sample attrition over time. Because the predictors of attrition are also predictors of child conduct problems, this sample is likely to under-represent children with the most severe difficulties, as well as family histories with the greatest increase in risk factors. Attrition weights designed by the survey team were used to address this problem, yet the results may still underestimate the importance of socio-economic risk factors. Fourth, the extent of the analyses were limited by the measures included in the multi-purpose longitudinal survey of a UK national cohort. As a result, this study was unable to examine important risk factors such as poor parenting. The use of SDQ, a clinical screening tool, may also be a limitation. The SDQ uses a narrowly defined pool of overt and covert items of conduct problems, which may reduce its reliability. Furthermore, the SDQ (Goodman et al., 2000), as well as CBCL (Lowe, 1998), are predictive of conduct disorder and other diagnoses; however, the conduct problem trajectories themselves are not clinical. As a result, the findings may differ from studies using multiple clinical measures to diagnose conduct disorder or oppositional defiant disorder (e.g., Frick, Cornell, Barry, Bodin, & Dane, 2003). In addition, there may be concerns regarding the generalizability of this UK cohort-based study to other international settings and contexts. Lastly, this study examined data only up to age 14 years and thus there is no evidence (yet) whether conduct problems persist throughout adolescence or into adulthood.

## Conclusion

Pathways into and out of conduct problems vary by changing circumstances experienced since infancy. This study addresses the gap in understanding how these developmental patterns diverge according to the timing and persistence of risk and whether there are differences across ecological domains. Using group-based modeling, this study examines variations in trajectories of conduct problems and associated child, family and socio-economic risk factors from early childhood to adolescence. The findings reveal not a single magic policy lever, but confirm that a number of different influences and processes may explain diverse pathways of conduct problems, offering insights into who and what might be targeted and when might be the most effective developmental window for intervention. The results emphasize the importance of pre-emptive support to reduce the likelihood of maternal smoking during pregnancy and buffer the effects of socio-economic deprivation in the first year of life. Interventions that improve children’s verbal skills and reduce emotional or other behavioral problems should also be undertaken in the earliest years, when conduct problems are first identified. Although many, if not most, of those identified at age 3 years are likely to desist in their problematic behavior by adolescence, such interventions may help ensure that they are not simply substituting their behavior with mental health difficulties or other problems. Early markers for those with adolescent-onset conduct problems indicate that this pathway is not a normative group of rebellious teens but vulnerable children who require additional support to manage the transition to early adolescence and beyond. School-based interventions aimed at reducing conduct problems, as well as supporting emotional health and boosting achievement, are likely to be effective for those showing persisting conduct problems as well as those with onset later in adolescence. Overall, this study provides robust empirical longitudinal evidence on developmental patterns of conduct problems manifesting from early childhood to adolescence. This draws attention to the importance of the timing and persistence of multiple risk factors, from the earliest ages, in shaping the divergent pathways of conduct problems and shows how the proximal correlates of conduct problems in adolescence have their roots in earlier social and family risks.
